# The relationship between stress, anxiety and eating behavior among Chinese students: a cross-sectional study

**DOI:** 10.3389/fpubh.2024.1466700

**Published:** 2024-10-09

**Authors:** Yulin Chai, Guoqi Fu, Yanxu Liu, Qi Song, Cailing Xue, Sheng Luo

**Affiliations:** School of Management, Shandong Second Medical University, Weifang, China

**Keywords:** stress, anxiety, eating behavior, college student, health psychology

## Abstract

**Background:**

The expansion of higher education and the growing number of college students have led to increased awareness of mental health issues such as stress, anxiety, and eating disorders. In China, the educational system and cultural expectations contribute to the stress experienced by college students. This study aims to clarify the role of anxiety as a mediator in the relationship between stress and eating behaviors among Chinese college students.

**Methods:**

This study utilized data from the 2021 Psychology and Behavior Investigation of Chinese Residents, which included 1,672 college students under the age of 25. The analysis methods comprised descriptive statistics, *t*-tests, Pearson correlation analyses, and mediation effect analysis.

**Results:**

The findings indicate that Chinese college students experience high levels of stress, with long-term stress slightly exceeding short-term stress. Both types of stress were positively correlated with increased anxiety and the adoption of unhealthy eating behaviors. Anxiety was identified as a significant mediator, accounting for 28.3% of the relationship between long-term stress and eating behavior (95% *CI* = 0.058–0.183). The mediation effect of short-term stress on eating behavior through anxiety was also significant, explaining 61.4% of the total effect (95% *CI* = 0.185–0.327).

**Conclusion:**

The study underscores the importance of stress management and mental health services for college students. It recommends a comprehensive approach to reducing external pressures, managing anxiety, and promoting healthy eating behaviors among college students. Suggestions include expanding employment opportunities, providing career guidance, enhancing campus and societal support for holistic development, strengthening mental health services, leveraging artificial intelligence technologies, educating on healthy lifestyles, and implementing targeted health promotion programs.

## 1 Introduction

The expansion of higher education has led to a steady increase in the global population of college students. Concurrently, there has been a concerning rise in the prevalence of mental health disorders among this demographic, including depression, anxiety, and eating disorders (1). This trend can be attributed to the propensity to develop unhealthy lifestyles and habits during college. Such behaviors include inadequate physical activity, excessive junk food consumption, initiation of smoking, heavy alcohol intake, irregular meal patterns, and the buildup of negative emotions (2, 3). Notably, the correlation between the accumulation of negative emotions and unhealthy eating behaviors among college students poses heightened health risks (4, 5).

In China, college students are particularly impacted by the nation’s higher education system and traditional mindsets, resulting in increased stress levels (6, 7). Stress, a fundamental biological concept indicating a disruption in homeostatic balance, encompasses systemic and localized responses and is relevant across psychological, physiological, social, and environmental domains (8). Physiologically, a moderate stress level can benefit college students by preparing them to anticipate and adapt to future challenges. However, chronic stress can elicit defensive responses, affecting emotions, behaviors, and physiological functions, potentially precipitating pathological states (9). Stress ranks among the top 10 health determinants, with excessive levels not only increasing the risk of high-risk behaviors but also adversely affecting psychological well-being and potentially inciting mental illnesses. These detrimental effects can accumulate over time (10). Studies have demonstrated a robust association between stress, depression, and anxiety among college students (11, 12). Excessive stress often presents as heightened anxiety and depressive symptoms. One study identified five distinct trajectories of anxiety and stress levels among college students: low and stable, decreasing then stable, increasing then decreasing, increasing, and decreasing then high (13). Furthermore, research has linked depression, stress, and the development of food addiction in college students (14). However, subsequent studies suggest that anxiety has a stronger association with eating behaviors than depression and stress, with anxiety being a more significant predictor of such behaviors (15).

According to Organismic Integration Theory (OIT), external motivators influence and regulate individual behavior (16). When college students face stress, their autonomy and sense of competence may be compromised, leading to internalized anxiety (13, 17). Anxiety, emerging as an internal response to unmet psychological needs, can prompt individuals to adopt specific eating behaviors as a regulatory mechanism, potentially evolving into unhealthy patterns over time. As college students represent a vital resource for national development, enhancing their physical and mental well-being is a societal priority (18). While existing research acknowledges the impact of negative emotions on college students’ eating behaviors, it often focuses on the direct effects of stress or anxiety, neglecting the potential influence of stress on anxiety levels. This study aims to investigate the mediating role of anxiety between stress and eating behaviors in Chinese college students, providing theoretical insights and practical strategies for predicting and addressing unhealthy eating behaviors through personalized health interventions.

## 2 Methods

### 2.1 Data sources

The data for this study was sourced from the Psychology and Behavior Investigation of Chinese Residents (PBICR) conducted in 2021. The survey spanned from July to September 2021. A multistage sampling approach was employed to select 120 cities, with quota sampling based on the findings of the seventh census of city residents, the final sample comprised 11,709 individuals. The PBICR 2021 received ethical approval from Jinan University (JNUKY-2021-018), and informed consent was secured from all participants. Focusing on school-age college students, the study identified participants whose current occupation was listed as “student” (3,314 individuals). We then excluded those outside the age range of 18–25 years (590 individuals) and further refined the sample by selecting only those pursuing a bachelor’s degree, resulting in a final sample of 1,672 college students within the 18–25 age range.

### 2.2 Measures

#### 2.2.1 Stress

Stress is defined as the disruption of an individual’s equilibrium by adverse factors, evoking a sense of threat (19). Participants reported their short-term (2 weeks) and long-term (1 year) stress in the PBICR, with stress levels gaged on a 0–6 scale, where a higher score indicates more significant perceived stress.

#### 2.2.2 Anxiety

Anxiety is regarded as a future-oriented emotional state marked by anticipatory cognitive, behavioral, and affective changes stemming from uncertainty about potential threats, with severe anxiety potentially causing significant distress (20). The PBICR utilized the Generalized Anxiety Disorder - 7 Item Scale (GAD-7) to assess respondents’ anxiety levels. The GAD-7 comprises seven items scored on a 4-point scale (0 = not at all, to 3 = nearly every day), with a total score ranging from 0 to 21, where higher scores denote increased anxiety (21). The GAD-7 has demonstrated robust construct validity and reliability within the college student demographic (22). In this study, Cronbach’s *α* was 0.965, and the Kaiser-Meyer-Olkin (KMO) measure was 0.947.

#### 2.2.3 Eating behavior

Eating behavior encompasses food management and the thoughts and feelings influencing food intake, including selection and procurement (23). The PBICR employed a short form of the Eating Behavior Scale (EBS-SF) to evaluate participants’ eating behaviors. The EBS-SF includes seven items rated on a 4-point scale (1 = strongly disagree, to 4 = strongly agree), with total scores ranging from 7 to 28, where higher scores suggest poorer eating behaviors (24). Ge et al.’s study (25) confirmed the validity and reliability of the Chinese version of the EBS-SF’s construct for assessing Chinese eating behaviors. In this study, Cronbach’s *α* was 0.865, and the KMO measure was 0.892.

#### 2.2.4 Demographic characteristics

To minimize confounding factors, demographic characteristics from the PBICR were incorporated, including gender, residence, only-child status, smoking and drinking habits, and Body Mass Index (BMI). College students were categorized into obese and non-obese groups based on established BMI cutoffs for males (≥ 23.53) and females (≥ 23.41) (26).

### 2.3 Statistical analysis

The study’s statistical analysis was conducted using SPSS 25.0. Continuous variables are presented as mean (SD), while categorical variables are expressed as percentages. Descriptive statistics were employed to summarize the data, and *t*-tests were used to compare group differences. Pearson correlation analysis was conducted to examine the relationships between variables. For mediation analysis and hypothesis testing, the PROCESS V4.2 macro was utilized, allowing us to assess the indirect effects and mediating role of variables (27). The significance level for all statistical tests was set at *α* = 0.05.

## 3 Results

### 3.1 Basic status

Among the 1,672 college students surveyed, most were female, resided in urban areas, were not only children, and had a minimal smoking prevalence. A small proportion engaged in drinking and the majority were not classified as obese. Statistically significant differences in anxiety were observed based on gender and smoking status, as well as in eating behaviors based on smoking, drinking, and obesity status (*p* < 0.05). Further details in Table 1. The mean anxiety score was (4.91 ± 4.99), approaching a mild level. The mean eating behavior score was (17.57 ± 4.53), slightly above the median. A positive and statistically significant correlation (*p* < 0.05) was identified between short-term stress, long-term stress, anxiety, and eating behavior. See Table 2 for details.

**Table 1:** Comparison of stress, anxiety, and eating behavior among college students with different characteristics (*N* = 1,672).

Variables	N (%)	Stress, Mean (SD)		Anxiety, Mean (SD)	EB, Mean (SD)
		Short term	Long term		
Gender					
Female = 0	1,022 (61.1)	2.99 (1.30)	3.24 (1.26)	4.68 (4.69)	17.61 (4.32)
Male = 1	650 (38.9)	3.12 (1.45)	3.32 (1.41)	5.27 (5.42)	17.50 (4.83)
*t*		−1.803	−1.287	−2.296	0.488
P		0.072	0.198	0.022	0.626
Residence					
Urban = 0	1,201 (71.8)	3.03 (1.38)	3.26 (1.31)	4.78 (5.02)	17.56 (4.60)
Rural = 1	471 (28.2)	3.08 (1.33)	3.30 (1.35)	5.23 (4.91)	17.58 (4.35)
*t*		−0.700	−0.493	−1.641	−0.099
P		0.484	0.622	0.101	0.921
Only child					
No = 0	1,001 (59.9)	3.06 (1.35)	3.25 (1.31)	4.99 (4.85)	17.44 (4.42)
Yes = 1	671 (40.1)	3.01 (1.38)	3.30 (1.33)	4.78 (5.19)	17.76 (4.68)
*t*		−0.830	−0.757	0.836	−1.411
P		0.407	0.449	0.403	0.159
Smoking					
No = 0	1,615 (96.6)	3.05 (1.36)	3.27 (1.31)	4.85 (4.94)	17.52 (4.51)
Yes = 1	57 (3.4)	2.86 (1.48)	3.14 (1.55)	6.60 (6.00)	18.86 (4.98)
*t*		1.023	0.754	−2.177	−2.197
P		0.306	0.451	0.034	0.028
Drinking					
No = 0	1,228 (73.4)	3.03 (1.35)	3.24 (1.31)	4.78 (4.99)	17.39 (4.47)
Yes = 1	444 (26.6)	3.07 (1.40)	3.35 (1.33)	5.26 (4.97)	18.05 (4.66)
*t*		−0.555	−1.523	−1.732	−2.604
P		0.579	0.128	0.083	0.009
Obese					
No = 0	1,363 (81.5)	3.02 (1.35)	3.26 (1.31)	4.80 (4.94)	17.14 (4.49)
Yes = 1	309 (18.5)	3.12 (1.42)	3.32 (1.37)	5.35 (5.18)	19.47 (4.20)
*t*		−1.084	−0.748	−1.734	−8.336
P		0.279	0.454	0.083	<0.001

EB, Eating behavior.

**Table 2:** Correlation analysis of stress, anxiety, and eating behavior among college students.

	M(SD)	1	2	3	4
1 Short-term stress	3.04 (1.36)	1.000			
2 Long-term stress	3.27 (1.32)	0.690^**^	1.000		
3 Anxiety	4.91 (4.99)	0.449^**^	0.371^**^	1.000	
4 Eating behavior	17.57 (4.53)	0.164^**^	0.125^**^	0.314^**^	1.000

^**^p < 0.01.

### 3.2 Mediating effects analysis

Existing literature suggests prolonged stress duration is more likely to elicit negative emotions and behavioral changes (28). More significant long-term stress can intensify the impact of short-term stress on mental health, with individuals experiencing long-term stress reporting a higher number of short-term stressors and increased psychological distress (29). Guided by these theories and findings, this study aimed to investigate the sequential mediating effects of long-term stress, short-term stress, anxiety, and eating behaviors. Eating behavior was considered the dependent variable, with long-term stress as the independent variable and short-term stress and anxiety as mediating variables. The study also controlled for variables significantly associated with eating behavior, such as smoking, excessive alcohol consumption, and obesity. The results indicate that long-term stress significantly and positively predicts short-term stress and anxiety (*p* < 0.05), short-term stress significantly and positively predicts anxiety (*p* < 0.05), and anxiety significantly and positively predicts eating behavior (*p* < 0.05). See Table 3 for the analysis.

**Table 3:** Mediating effects of short-term stress and anxiety.

		B	*t*	P	*β*	R^2^	P
Eating behavior	Long-term stress	0.414	5.069	<0.001	0.121	0.058	<0.001
	Smoke	1.015	1.686	0.092	0.041		
	Drink	0.376	1.517	0.129	0.037		
	Obese	2.248	8.081	<0.001	0.193		
Short-term stress	Long-term stress	0.714	38.905	<0.001	0.690	0.477	<0.001
	Smoke	−0.083	−0.616	0.538	−0.011		
	Drink	−0.036	−0.639	0.523	−0.012		
	Obese	0.057	0.911	0.362	0.016		
Anxiety	Long-term stress	0.444	3.906	<0.001	0.117	0.215	<0.001
	Short-term stress	1.348	12.285	<0.001	0.369		
	Smoke	1.943	3.206	0.001	0.071		
	Drink	0.213	0.854	0.393	0.019		
	Obese	0.326	1.163	0.245	0.025		
Eating behavior	Long-term stress	−0.047	−0.435	0.664	−0.014	0.136	<0.001
	Short-term stress	0.126	1.152	0.249	0.038		
	Anxiety	0.264	11.324	<0.001	0.291		
	Smoke	0.542	0.936	0.350	0.022		
	Drink	0.337	1.417	0.157	0.033		
	Obese	2.135	8.000	<0.001	0.183		

**Figure 1:**
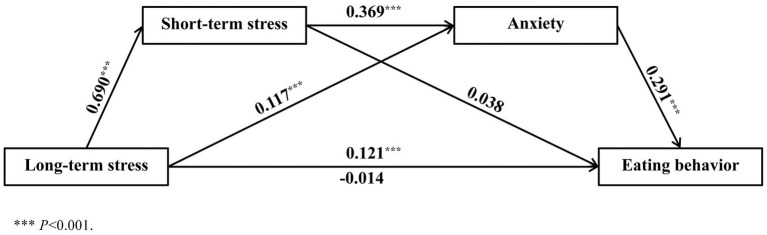
Mediating model of the effect of stress on eating behavior among college students.

The significance tests were conducted using the bootstrap method, which involved generating 5,000 random resamples from the original data. A mediating effect was considered significant if the 95% confidence interval (CI) derived from these resamples did not include zero. The study findings revealed that the direct effect of long-term stress on eating behavior (95% *CI* = −0.261–0.166) and the mediating effect of short-term stress was insignificant (95% *CI* = −0.061–0.251). However, anxiety emerged as a significant mediator (95% *CI* = 0.058–0.183), accounting for 28.3% of the total effect. Furthermore, the sequential mediation involving short-term stress and anxiety was also significant (95% *CI* = 0.185–0.327), explaining 61.4% of the total effect. Collectively, short-term stress and anxiety jointly functioned as full mediators in the relationship between long-term stress and eating behavior (95% *CI* = 0.314–0.616). Refer to Table 4 and Figure 1 for visual representation.

**Table 4:** Significance tests for mediating effects.

Effect	Paths	B	95% *CI*	Ratio (%)
Direct effect	Long-term stress→Eating behavior	−0.047	−0.261–0.166	
Indirect effect	Long-term stress→Short-term stress→Eating behavior	0.090	−0.061–0.251	
	Long-term stress→Anxiety→Eating behavior	0.117	0.058–0.183	28.3
	Long-term stress→Short-term stress→Anxiety→Eating behavior	0.254	0.185–0.327	61.4
Total indirect effect		0.462	0.314–0.616	
Total effect		0.414	0.254–0.575	

## 4 Discussion

### 4.1 College students perceive more long-term stress than short-term stress

This study indicates that Chinese college students report slightly higher levels of long-term stress compared to short-term stress, with both surpassing the median. This pervasive stress is consistent with prior research (10). The highly competitive nature of China’s education system, especially at the higher education level, along with the academic and employment challenges, are contributing factors (30). Pursuing high grades, engaging in extracurricular activities, and internships to boost competitiveness and concerns about future employment can amplify stress levels (31). Long-term employment stress may thus overshadow temporary academic stress.

### 4.2 Differences in anxiety and eating behaviors among college students

Gender and smoking status were found to significantly influence anxiety levels among college students, corroborating previous findings (32, 33). Smoking, often used as a coping mechanism, is linked to higher anxiety levels (34). Interestingly, this study found that males exhibited higher anxiety levels, possibly due to measurement methods, sample size, and gender equality initiatives in China’s higher education (35). Efforts toward gender equality may have mitigated anxiety among female students.

Additionally, students who smoke, drink, and are obese displayed poorer eating behaviors, in line with prior research (36–38). Unhealthy habits associated with these conditions can lead to poor eating behaviors, potentially due to reduced self-regulation and inclinations toward indulgent food choices (39). Physiological changes from such habits may also affect appetite, influencing eating behaviors (3).

### 4.3 Relationships between stress, anxiety, and eating behaviors among college students

The study’s findings reveal that both short-term and long-term stress impact anxiety and eating behavior among college students, echoing previous studies (40, 41). Elevated stress levels correlate with increased anxiety and poor eating behaviors. Stress can accumulate, leading to emotional distress and disruptions in emotional state and self-control, thus increasing anxiety (42). Moreover, stress can alter eating behaviors, with higher stress often leading to increased consumption (43). Higher anxiety levels are also associated with unhealthy eating behaviors and disorders (44), highlighting the complex interplay among stress, anxiety, and eating behaviors.

### 4.4 Mediating effects of short-term stress and anxiety between long-term stress and eating behaviors among college students

The mediating effects analysis shows that anxiety mediates the relationship between long-term stress and eating behavior, in agreement with previous studies (45). When subjected to stress, individuals become more susceptible to emotional distress and the experience of negative emotions, such as anxiety or depression. In such circumstances, the propensity for unhealthy eating behaviors escalates, potentially culminating in overeating or the selection of unhealthy food options (46). Moreover, the study identified a full chain-mediating effect for both short-term stress and anxiety. This suggests that college students confronting persistent stress are at a higher risk of developing a stress response that is internalized as anxiety, thereby precipitating uncontrolled eating behaviors (23). This phenomenon could originate from the fact that those who suffer from extended stress are more likely to face compromised coping mechanisms, reduced emotional regulation capabilities, and a negative cognitive bias. These elements can lead to the development of stress sensitivity, causing individuals to perceive escalating stress levels, which in turn can amplify anxiety and precipitate unhealthy eating behaviors.

Additionally, despite being minimal and statistically insignificant in this study, long-term stress appeared to exert a modest inhibitory effect on unhealthy eating behaviors. Some researchers have posited that an optimal level of stress might aid college students in adapting to challenges (9). However, this potential adaptive effect may not have been evident in this study due to the higher levels of long-term stress experienced by Chinese college students. Future research could benefit from in-depth analysis, employing control group experiments to elucidate these dynamics further.

### 4.5 Implications

The study’s findings carry substantial implications for college students’ mental and physical health. They underscore the interconnected relationship between stress, anxiety, and eating behaviors, providing valuable insights into the mechanisms at play. In light of these findings, a multifaceted approach is recommended to mitigate external stress, internal anxiety, and unhealthy eating behaviors among college students. Firstly, the government and universities should address long-term stress related to employment prospects by expanding job opportunities and offering robust career guidance. Efforts to reduce short-term stress within higher education include cultivating a campus and social environment that promotes holistic student development. Secondly, educational institutions should enhance mental health services. This includes offering courses fostering positive thinking and accessible psychological counseling to support students dealing with stress or anxiety. Leveraging advancements in artificial intelligence, institutions can employ specialized AI platforms like HealthBuddy, Florence, and Buoy Health or utilize data analysis services to detect psychological and psychiatric disorders (47). Given their high level of education, college students are well-positioned to benefit from these technologies and adopt proactive measures to manage stress and anxiety, thereby influencing healthier eating behaviors. Lastly, it is imperative to initiate educational campaigns promoting healthy lifestyles. Advocating for people-centered health communication strategies that prioritize assessing the health needs of university students is essential (48). Based on these assessments, targeted campaigns should encourage smoking cessation, moderate alcohol consumption, regular physical activity, and a balanced diet. These initiatives are critical in preventing the onset of anxiety and unhealthy eating patterns among college students.

### 4.6 Limitations

This study has several limitations. The use of cross-sectional data limits the ability to establish causality, and self-reported measures of stress may introduce bias. The data’s cultural specificity to China also suggests caution in generalizing the findings to other contexts.

## 5 Conclusion

This study provides valuable insights into the interplay between stress, anxiety, and eating behaviors among college students. The results demonstrate a positive correlation between both short-term and long-term stress and the escalation of anxiety and unhealthy eating behaviors within this demographic. Furthermore, anxiety is revealed to be a significant mediator in the relationship between stress and eating behaviors.

In light of these findings, devising and implementing holistic mental health interventions is imperative. These interventions should concentrate on alleviating stress and anxiety levels. They are designed to empower college students to develop healthier eating habits, reduce associated health risks, and foster their overall health.

## Data Availability

Publicly available datasets were analyzed in this study. This data can be found at: Psychology and Behavior Investigation of Chinese Residents (https://www.x-mol.com/groups/pbicr).
